# Autoimmune inflammation as a key risk factor for heart failure with preserved ejection fraction: the different types of inflammation driving to HFpEF

**DOI:** 10.3389/fmed.2025.1557312

**Published:** 2025-10-15

**Authors:** Elisa Gremese, Dario Bruno, Simone Perniola, Jacopo Ceolan, Gianfranco Ferraccioli

**Affiliations:** ^1^Department of Biomedical Sciences, Humanitas University, Pieve Emanuele, Italy; ^2^Rheumatology and Clinical Immunology, IRCCS Humanitas Research Hospital, Rozzano, Italy; ^3^Rheumatology Unit, Department of Precision and Regenerative Medicine and Ionian Area (DiMePRe-J), University of Bari, Bari, Italy; ^4^Department of Engineering for Innovative Medicine, University of Verona and Azienda Ospedaliera Universitaria Integrata Verona, Verona, Italy; ^5^Department of Medicine-Catholic University of the Sacred Heart, Fondazione Policlinico Gemelli IRCCS, Rome, Italy

**Keywords:** inflammation, autoimmunity, endothelial dysfunction, diastolic dysfunction, heart failure with preserved ejection fraction

## Abstract

**Importance:**

Heart failure with preserved ejection fraction (HFpEF), defined by an ejection fraction >50%, has emerged as the most prevalent form of heart failure at the community level. Multiple comorbidities, including diabetes, hypertension, obesity, atrial fibrillation, renal diseases, and autoimmune conditions, have been linked to its development. These conditions share common pathways involving oxidative stress, metabolic dysregulation, ischemia, and a chronic inflammatory milieu.

**Observations:**

Patients with autoimmune diseases such as rheumatoid arthritis (RA), systemic lupus erythematosus (SLE), and systemic sclerosis (SSc) exhibit an increased risk of developing HFpEF, often through mechanisms involving chronic inflammation and endothelial dysfunction, which precede the clinical manifestation of HFpEF. Clinical studies have demonstrated that the risk of developing HFpEF exists independently of traditional cardiovascular risk factors, underscoring the pivotal role of chronic inflammation and autoimmunity as key contributors to its pathogenesis.

**Conclusions and relevance:**

The translational implication is that the distinct inflammatory pathways driving these autoimmune diseases (e.g., myeloid-T cells and T-B cell-mediated inflammation in RA, and B cell-driven inflammation in SLE and SSc) should become personalized therapeutic targets to prevent HFpEF progression. Early intervention with novel therapies, such as sodium-glucose cotransporter type 2 (SGLT2) inhibitors, could be crucial in managing these patients during the early disease stages. Additionally, the H2FPEF score should be routinely employed to facilitate early diagnosis and risk stratification, providing a robust framework for personalized management strategies.

## Introduction

Heart failure with preserved ejection fraction (HFpEF) has emerged as a leading cause of mortality among heart failure patients (1). According to the current guidelines of the American Heart Association/American College of Cardiology and the European Society of Cardiology, the diagnosis of HFpEF is based on three primary criteria: 1. the presence of signs and symptoms consistent with heart failure; 2. a preserved left ventricular ejection fraction (LVEF ≥50%); and 3. objective evidence of impaired left ventricular (LV) diastolic function (2). Estimates suggest that at least 50% (range 44–72%) of all heart failure cases occur with preserved ejection fraction (3).

Community-based data from Olmsted County indicate that only 16% of HFpEF patients had a prior myocardial infarction, compared to 28% of those with heart failure with reduced ejection fraction (HFrEF). Additionally, coronary heart disease accounted for 29% of deaths in HFpEF patients compared to 43% in HFrEF patients (4). These findings suggest that coronary artery disease plays a less dominant role in HFpEF, while myocardial disease appears to be more prevalent. Between 2000 and 2010, the proportion of HFpEF among new heart failure cases in Olmsted County increased from 48 to 52%, with women being affected twice as often as men. Furthermore, over this decade, the incidence of HFpEF showed a smaller decline compared to HFrEF (−27 versus −61%, respectively) (5).

HFpEF is generally characterized by older age, female predominance, and a higher prevalence of atrial fibrillation, with lower rates of stroke and coronary artery disease (1). Its global prevalence is rising, driven by both traditional risk factors (i.e., obesity, diabetes, hypertension, smoking, metabolic syndrome, renal failure, anemia), and emerging pathophysiological mechanisms, including diastolic dysfunction, endothelial dysfunction, microvascular damage, and systemic low-grade inflammation that promotes myocardial remodeling (3, 6). Oxidative stress and fibrosis are also recognized as critical contributors to the disease’s pathogenesis (7).

Inflammation plays a pivotal role in the development of heart failure, potentially contributing differently to its various subtypes, with evidence highlighting a specific association between the interleukin-6 (IL-6)/C-reactive protein (CRP) pathway and the pathogenesis of HFpEF (8). In inflammatory and autoimmune rheumatologic diseases, HFpEF remains underrecognized, despite evidence suggesting that its development may be driven by distinct autoimmune and inflammatory mechanisms specific to each condition.

Therefore, in this review, we focus on evidence from the past two decades (2004–2024) exploring the intersection of HFpEF and three autoimmune diseases: rheumatoid arthritis (RA), systemic lupus erythematosus (SLE), and systemic sclerosis (SSc). Specifically, we conducted a literature search using PubMed and Scopus, covering the years 2004–2024. Search terms included “HFpEF,” “diastolic dysfunction,” “autoimmune,” “rheumatoid arthritis,” “SLE,” and “systemic sclerosis.” We included english-language studies focusing specifically on HFpEF in the context of autoimmune diseases, ultimately identifying five studies in RA, seven prospective studies overall, and one observational study with relevant clinical data. We excluded studies that did not clearly distinguish between HFpEF and HFrEF, or that lacked primary data on cardiovascular outcomes.

## Endothelial dysfunction, chronic inflammation, diastolic dysfunction, and HFpEF: experimental models

While not all diastolic dysfunctions (DD) progress to HFpEF, all HFpEF cases exhibit DD (9). Understanding the pathophysiology of DD is therefore crucial to elucidate its progression to heart failure. An ideal murine model of HFpEF should present specific characteristics, such as exercise intolerance, pulmonary edema, concentric cardiac hypertrophy, and a preserved EF > 50% (10). Among the proposed models, three particularly emphasize the link between DD and inflammation.

**Figure 1:**
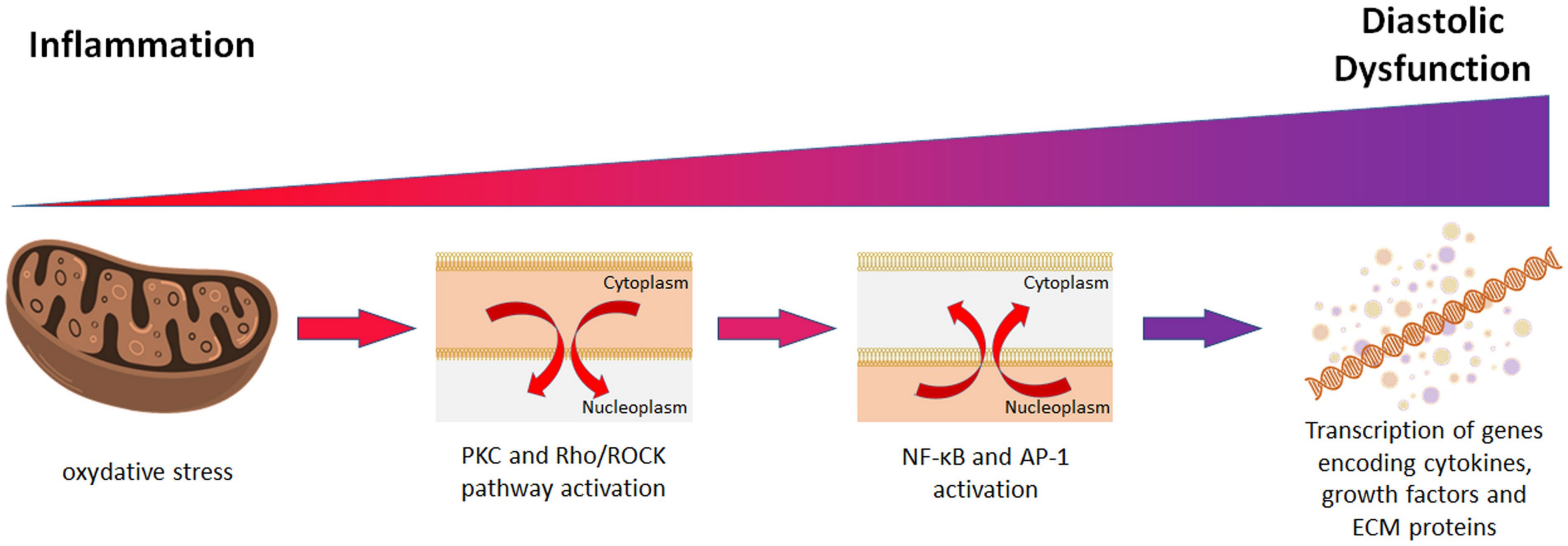
During inflammatory response, in the mytochondrial environment, oxidative stress activates PKC and Rho/ROCK pathway, subsequently triggering cellular NF-κB and AP-1, which drive inflammatory cascades. Thus, contributes to the development of coronary vasospasm, hypertension, and myocardial remodeling, ultimately resulting in diastolic dysfunction. PKC, protein kinase C; NF-κB, nuclear factor kappa light chain of B cells; AP-1, activator protein-1; ECM, extracellular matrix.

In Goto-Kakizaki (GK) rats, a prediabetic model with insulin deficiency, DD originates in the myofilaments. Synchrotron radiation small-angle X-ray scattering (SAXS) on beating hearts revealed displacement of myosin heads from actin filaments during diastole, along with impaired relaxation and cross-bridge dynamics (11, 12). Mitochondrial oxidative stress and elevated activity of protein kinase C (PKC) and Rho kinase (ROCK) increase cardiomyocyte stiffness and passive tension, ultimately promoting DD (13). Oxidative stress acts as a secondary messenger, activating PKC (14) and the Rho/ROCK pathway (15), which in turn trigger NF-κB and AP-1 activation. These pathways promote cytokine and growth factor transcription, extracellular matrix (ECM) remodeling, vasospasm, hypertension, and myocardial remodeling (16, 17) (Figure 1).

Notably, GK rats showed elevated myocardial IL-6, TGF-β1, and Nox2 (a ROS-producing enzyme). Despite these changes, eNOS and NO-mediated vasodilation were preserved. These findings establish oxidative stress and inflammation as central mechanisms driving DD and endothelial dysfunction (13, 17). Likewise, in women with ischemia but no coronary artery disease, oxidative stress has been linked to DD (18).

Diabetes further contributes to DD via chronic low-grade inflammation, termed “metabolic inflammation” (19). Once DD develops, its association with ED becomes evident (20, 21), and ED has emerged as a promising therapeutic target in heart failure (22).

Additional validated models of DD include the SAUNA model (salty water, unilateral nephrectomy, aldosterone) and an aging murine model. In both, increased hematopoiesis correlates with macrophage recruitment and elevated ROS production. These macrophages secrete TGF-*β* and IL-10, promoting fibroblast activation and ECM synthesis (e.g., type I collagen, *α*-SMA) (23, 24).

Resident cardiac macrophages (RCMs), classified as CCR2 + or CCR2-, play differential roles. CCR2- macrophages aid repair and angiogenesis (25), while CCR2 + macrophages fuel inflammation through IL-1β and Nlrp3 activation, contributing to adverse remodeling (26). In failing human hearts, CCR2 + cells dominate, enriched in NF-κB, IL-6, and STAT3 pathways (27, 28). These cells also express oncostatin M (OSM), known to inhibit myoblast differentiation, especially after ischemic injury (27). Single-cell RNA-seq studies confirmed their pro-inflammatory role (28).

Thus, even conditions like hypertension and aging contribute to cardiac injury and DD, largely through inflammation-driven mechanisms.

**Figure 2:**
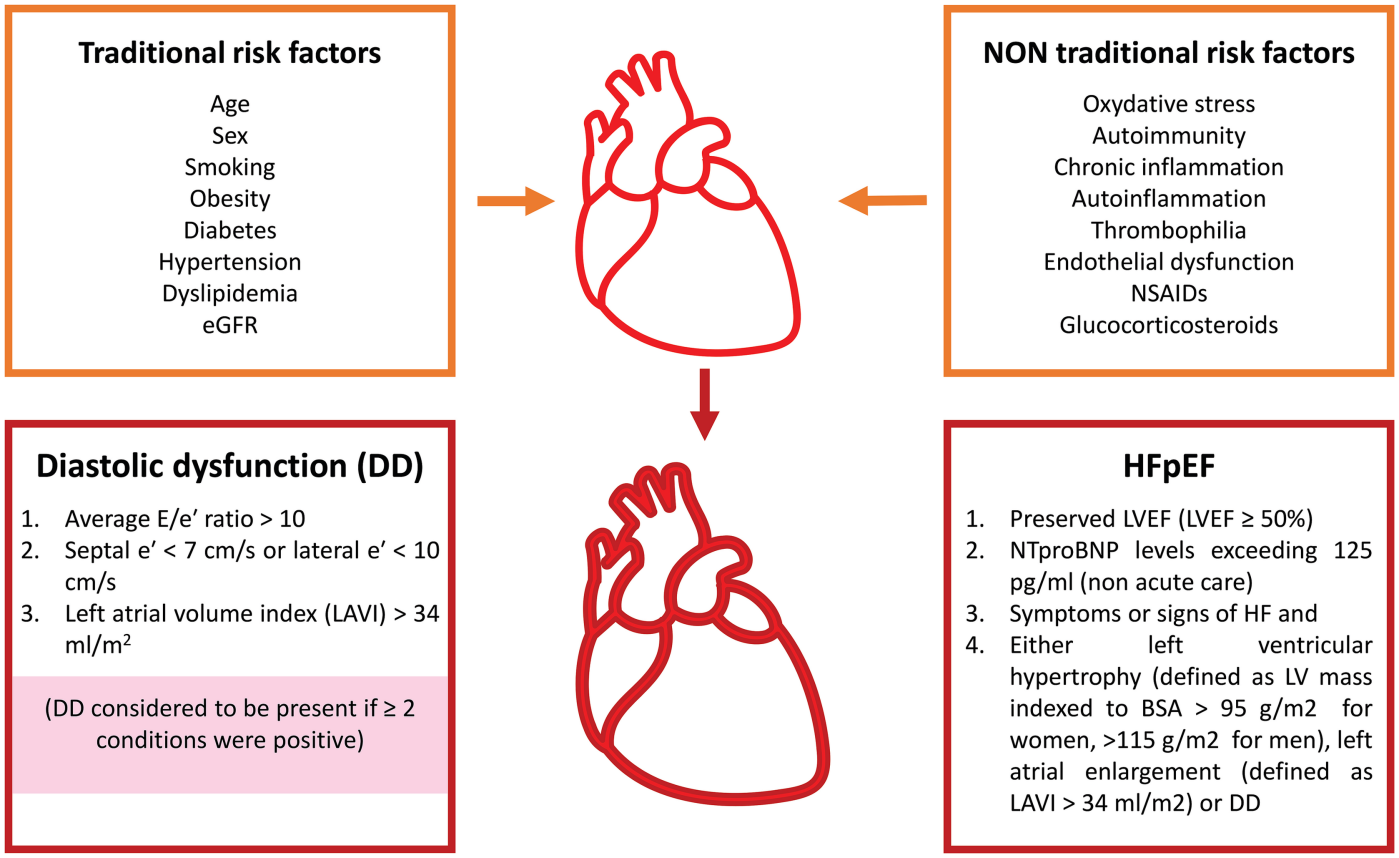
Traditional and non-traditional risk factors leading to diastolic dysfunction and to heart failure with preserved ejection fraction. eGFR, estimated glomerular filtration rate; NSAIDs, non-steroidal anti-inflammatory drugs; HFpEF, heart failure with preserved ejection fraction; LVEF, left ventricular ejection fraction; NTproBNP, N-terminal pro–B-type natriuretic peptide; BSA, body surface area.

In conclusion, the pathophysiology of HFpEF encompasses cardiomyocyte stiffness, fibrosis, microvascular dysfunction, oxidative stress, and chronic inflammation. As stated by Paulus and Tschope (29), all comorbidities associated with HFpEF appear to converge on a shared inflammatory axis that sustains myocardial dysfunction (Figure 2).

This section emphasizes that inflammation is a unifying mechanism across diverse HFpEF models and sets the stage for exploring human clinical phenotypes.

## HFpEF human phenotypes

These experimental insights highlight how inflammation initiates and perpetuates the pathophysiology of HFpEF and justify exploration of clinical phenotypes linked to such mechanisms. The relationship between HFpEF and comorbidities is well-documented beyond aging (30, 31). Across cohorts, approximately 45% of HFpEF patients have diabetes (32), 80% in the US are obese (33), 40–60% present with atrial fibrillation/flutter (34, 35), 55% have hypertension (36–38), and 26–49% have renal disease (39, 40). These comorbidities collectively create a low-to-moderate inflammatory state. Combined with neurohormonal, metabolic, and ischemic factors, this milieu promotes myocardial stiffness via oxidative stress, ischemia, and inflammation (Table 1).

**Table 1:** Experimental models and *in vivo* human phenotypes of endothelial and diastolic dysfunction ending up to HFpEF.

Experimental models	
GOTO-KAKIZAKI Diabetes rat model (Insulin deficient- increased PKC and ROCK activity- Cardiomyocyte stiffening) (11, 12)	Diastolic dysfunction precedes endothelium dysfunction
SAU-NA Hypertensive mouse model (unilateral nephrectomy, chronic exposure to aldosterone and accelerated mortality—hypertensive model—increased recruitment of macrophages CCR2+) (23)	Diastolic dysfunction followed by cardiomyopathy and accelerated mortality
AGING Mouse model (increase in left ventricular mass, interstitial fibrosis, with high expression of TGFb and IL10 and CCR2 + macrophages) (24)	Diastolic dysfunction, cardiomyocites hypertrophy and stiffness, microvascular dysfunction

Human phenotypes (age as the major risk factor)	
DIABETES (pathophysiologic mechanisms: alteration in sodium handling; increased volume overload; release of pro-inflammatory cytokines; endothelial and diastolic dysfunction) (32)	45% of HFpEF have diabetes
OBESITY (pathophysiologic mechanisms: volume overload; endothelial and diastolic dysfunction; biventricular remodeling; impaired pulmonary vasodilation; systemic inflammation) (33)	80% of HFpEF in US are obese
ATRIAL FIBRILLATION and FLUTTER (pathophysiologic mechanisms: widespread endothelial dysfunction; oxidative stress; microvascular inflammation with increased CRP levels; atrial and ventricular fibrosis) (35)	40–60% of HFpEF have atrial fibrillation or flutter
HYPERTENSION (pathophysiologic mechanisms: coronary microvascular endothelial dysfunction; increased afterload on left ventricle; ventricular hypertrophy; diastolic dysfunction; systemic inflammation) (37)	55% of patients with HFpEF have hypertension
CHRONIC RENAL DISEASES (pathophysiologic mechanisms: endothelial dysfunction, inflammation and systemic and renal fibrosis are mutual consequences of diabetes, hypertension and dyslipidaemia, which can also be drivers of cardiorenal syndrome) (40)	HFpEF patients: 26–49% have renal disease

Understanding these phenotypes helps contextualize the relevance of inflammation in HFpEF and paves the way to analyze autoimmune conditions in the following sections.

## Chronic inflammation, autoimmunity, and the heart

Understanding the role of systemic inflammatory burden across populations helps translate experimental evidence into clinical relevance.

Chronic heart inflammation, unlike acute myocarditis, is typically driven by autoimmune diseases, which vary in inflammatory load and vascular involvement. Analyzing cardiovascular comorbidities in these conditions provides valuable insights into how chronic inflammation contributes to HFpEF.

Several studies have shown that the risk of acute myocardial infarction (AMI) in rheumatoid arthritis (RA) rivals that of type 2 diabetes (41), and that heart failure (HF) risk is doubled in RA compared to the general population (42). The QRISK 3 algorithm now includes RA and systemic lupus erythematosus (SLE) in its 10-year cardiovascular risk estimation (43). Additionally, persistent inflammation—as measured by high-sensitivity CRP—has been shown to better predict cardiovascular events and mortality than LDL cholesterol in statin-treated patients (44).

Notably, the Reynolds score used in women also incorporates hsCRP, linking inflammation and cardiovascular risk. CRP is strongly associated with endothelial dysfunction (ED) in hyperlipidemic individuals (45), reinforcing the tight interplay among inflammation, lipids, and endothelial damage.

Together, these observations build a strong rationale for focusing on vascular inflammation as a shared pathway driving HFpEF in autoimmune diseases.

## Autoimmunity, chronic inflammation, and diastolic dysfunction

Diastolic dysfunction (DD) affects approximately 28.1% of the general population (46), where it independently predicts mortality and heart failure (47, 48). In autoimmune diseases, DD is even more prevalent and strongly associated with disease features.

For example, in RA, DD was observed in 31% of patients and linked to disease duration and elevated IL-6 levels (49). Premenopausal RA patients showed an even higher prevalence (47%) compared to age-matched controls (26%), with CRP being the strongest independent predictor (50).

In PsA, DD prevalence reached 38%, associated with older age and hypertension (51). In SSc, DD affected 35% of patients, regardless of whether disease was limited or diffuse, and correlated with Raynaud’s duration (52).

In SLE, 39% had DD independent of disease activity (SELENA-SLEDAI), with disease duration being the strongest determinant, while the Framingham score proved unreliable (53). Anti-cardiolipin antibodies, especially LAC, predicted worse DD progression (54).

Similarly, in IBD, DD was associated with reduced coronary flow reserve (CFR), an indicator of microvascular function (55), and cardiovascular risk has been recognized by expert panels (56).

These findings consistently show that autoimmune and chronic inflammatory diseases are strong contributors to DD, reinforcing the importance of cardiovascular monitoring in these patients.

## Endothelial dysfunction in autoimmune-chronic inflammatory diseases: a screening of diastolic dysfunction?

The 2013 paradigm by Paulus and Tschöpe (29) proposed that cardiovascular risk factors induce systemic inflammation, which impairs endothelial and coronary microvascular function, ultimately leading to HFpEF. This is supported by histological evidence of microvascular rarefaction and NOX2 expression in macrophages from HFpEF patients (57), as well as high prevalence of vascular dysfunction in this condition (58). Accordingly, autoimmune diseases frequently exhibit ED. Specifically:

1. RA: impaired response to acetylcholine, reversible with TNF-*α* blockade; long-term improvement requires disease remission (59, 60).
2. SSc: ED reversible with endothelin A receptor antagonism, but not with nitroprusside (61).
3. SLE: reduced FMD, worsened by comorbidities (62, 63).
4. PMR: FMD remained low even after 6 months of treatment, inversely correlated with CRP (64).

Normal FMD is ~6.4%, with age-related decline (65); standardized protocols now enable its use as a biomarker (66). Moreover, prospective studies show that ED predicts DD progression (67), and DD precedes HFpEF (48). Hence, maintaining control of systemic inflammation (as in RA and SLE) is essential (60, 68).

All together, these data support the concept of ED as an early and actionable marker in the prevention of HFpEF among patients with chronic autoimmune inflammation.

## HFpEF in rheumatoid arthritis, lupus and systemic sclerosis

While DD and ED are well-documented in autoimmune diseases, the clinical burden of HFpEF is only recently emerging as a distinct phenotype. Multiple studies from 2008 to 2024 have demonstrated that HFpEF is the dominant HF subtype in these populations (69–73) (Table 2). In RA, one-year mortality after HF diagnosis was 35%, compared to 19% in controls (69), and incidence ranged from 2.5 to 8.2% across cohorts (70–72). These risks remained stable over decades and were linked to disease activity.

**Table 2:** Clinical evidence of HFpEF in autoimmune diseases.

Study	Disease	Key findings	Notable observations
Davis et al. (69)	RA	35% 1-year mortality after HF vs. 19% in controls	High mortality burden in RA-related HF
Huang et al. (70)	RA	8.2% developed HF over 10.7 years	Long-term CV risk in RA
Mantel et al. (71)	RA	2.5% HF incidence over 5 years	Modest but relevant incidence
Myasoedova et al. (72)	RA	Stable HF prevalence over 30 years	Persistent CV burden despite treatment evolution
Ahlers et al. (73)	RA	HF incidence: 4.87 vs. 3.96 per 1,000 person-years	Higher chronic inflammatory load linked to HF
Athero-APS Study (74)	APS/SLE	HFpEF prevalence: 6.3% (carriers) to 27.8% (SLE-APS)	Severity-dependent CV risk escalation
Prasada et al. (75)	SSc, SLE, RA	HR for HF: 7.26 (SSc), 3.15 (SLE), 1.39 (RA)	Significant HF risk across diseases
Nomigolzar et al. (76)	SLE	0.61% of 10 M HF cases had SLE; higher in-hospital mortality	Increased pericardial complications
Oliveira et al. (77)	SSc	27% met HFpEF criteria	Age, AF, and ILD were key predictors
Rivera et al. (78)	ACIDs	70.5% with HF had HFpEF	Higher rate than general population
Tada et al. (79)	ACIDs	3x increased risk of death/hospitalization in HFpEF with ACID	Poorer prognosis vs. non-ACID patients

RA, rheumatoid arthritis; APS/SLE, antiphospholipid syndrome/systemic lupus erythematosus; SSc, systemic sclerosis; ACIDs, autoimmune and chronic inflammatory diseases; HF, heart failure; HFpEF, heart failure with preserved ejection fraction; AF, atrial fibrillation; ILD, interstitial lung disease.

Similarly, HF incidence was higher in RA (4.87/1,000 person-years vs. 3.96 in controls) (73). In other autoimmune diseases, HFpEF also emerged as the predominant phenotype. For instance, the Athero-APS study showed an increasing gradient of HFpEF prevalence from asymptomatic aPL carriers (6.3%) to full-blown SLE-APS (27.8%) (74). Large population studies confirmed that HF risk is markedly elevated in SSc, SLE, and RA (75), with worse in-hospital outcomes for SLE patients (76). In SSc, 27% met HFpEF criteria, and interstitial lung disease was a key predictor (77). Up to 70.5% of patients with autoimmune HF had the preserved EF phenotype (78).

Interestingly, RA patients on biologics were more likely to recover EF (78), but those with autoimmune comorbidities had a 3x higher risk of mortality or hospitalization (79). The underlying inflammatory drivers differ: RA involves myeloid–T and T–B cell inflammation (80, 81), SLE and SSc involve B-cell-mediated pathways (82–85).

Thus, therapies should reflect this heterogeneity: IL-6 blockers show promise in ischemic damage (86), T cell costimulation blockade prevents age-related dysfunction (87), and B-cell depletion has improved dilated cardiomyopathy (88).

This highlights the need for a personalized, inflammation-targeted approach in preventing and managing HFpEF in autoimmune disease.

## Evidence and perspectives

Controlling inflammation has emerged as a crucial strategy for improving diastolic dysfunction and potentially preventing HFpEF. Animal studies have offered compelling evidence supporting this approach. In a model of HFpEF using DAHL/SS salt-sensitive hypertensive rats, the administration of colchicine significantly improved survival, reduced cardiac dysfunction, and diminished oxidative stress and inflammatory cell infiltrates (89). These findings suggest the potential efficacy of colchicine, with human trials expected to provide further clarification (90).

**Figure 3:**
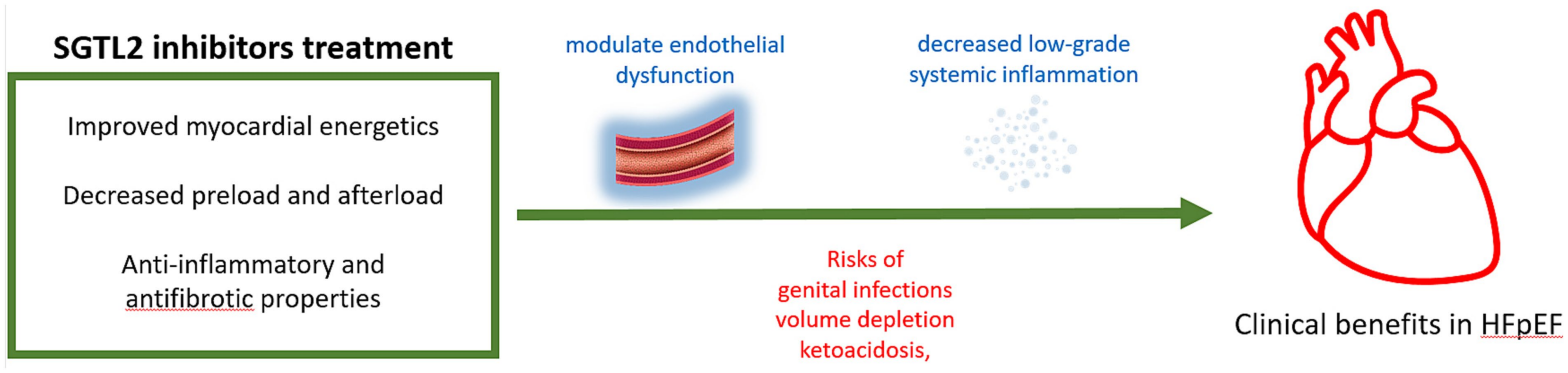
SGLT2 inhibitors have demonstrated clinical benefits in HFpEF, reducing the risk of heart failure hospitalization and cardiovascular death by modulating endothelial dysfunction and low-grade systemic inflammation, leading to improve myocardial energetics, decrease preload and afterload, and thought anti-inflammatory and antifibrotic properties. Nonetheless, adverse effects include genital infections, volume depletion, and ketoacidosis. SGLT2, sodium-glucose cotransporter 2; HFpEF, heart failure with preserved ejection fraction.

Among the most promising emerging therapies, sodium-glucose cotransporter 2 (SGLT2) inhibitors have demonstrated clinical benefits in HFpEF, particularly in patients with comorbid conditions such as type 2 diabetes and obesity. Results from large randomized trials, including EMPEROR-Preserved (91) and DELIVER (92), showed that treatment with empagliflozin or dapagliflozin significantly reduced the risk of heart failure hospitalization and cardiovascular death. These effects are thought to arise from improved myocardial energetics, decreased preload and afterload, and anti-inflammatory as well as antifibrotic properties. While data specifically addressing autoimmune populations are currently lacking, the potential of SGLT2 inhibitors to modulate endothelial dysfunction and low-grade systemic inflammation suggests they may also benefit patients with autoimmune-driven HFpEF. Nonetheless, clinicians should be cautious of adverse effects, including genital infections, volume depletion, and ketoacidosis, particularly in elderly or non-obese individuals (Figure 3). Further studies are needed to explore the safety and efficacy of these agents in this specific subgroup.

Plasma IL-6 has been a focal point of recent research, with its levels showing a strong predictive value for HFpEF but not for HFrEF in the PREVEND cohort—a prospective study of 961 participants. This association persisted even after adjusting for key risk factors, suggesting IL-6 as a potential target for novel therapeutic strategies (93). Supporting this, IL-6 was found to be an independent predictor of all-cause mortality in hospitalized HFpEF patients, even after accounting for B-type natriuretic peptide (BNP) levels (94). Furthermore, tocilizumab, an IL-6 receptor antagonist, demonstrated improvements in left ventricular ejection fraction in rheumatoid arthritis patients without overt cardiac symptoms, reinforcing the potential benefits of targeting IL-6 (95).

However, the results of targeting inflammation in HFpEF have been mixed. Anakinra, an IL-1 receptor antagonist targeting IL1α/β, failed to improve cardiac function in obese HFpEF patients, despite successfully lowering CRP and NT-proBNP levels (96). Similarly, the CANTOS trial, which investigated canakinumab (an anti-IL1β therapy), found that higher IL-6 levels 3 months post-initiation were associated with a substantial increase in major adverse cardiovascular events (MACE) and all-cause mortality (97), complicating the role of IL-1β inhibition in this context.

The link between inflammation and NT-proBNP levels provides additional insight. Among participants in the MESA study, IL-6 levels were significantly correlated with NT-proBNP levels, although it remains unclear whether these increases directly reflect the risk of incident HFpEF (98).

Of particular interest is the emerging evidence regarding IL-17. A preliminary study indicated that secukinumab, an IL-17A inhibitor, improved inflammation and diastolic dysfunction, which was present in nearly 39% of patients (99). If confirmed, this finding is especially significant given the central role of IL-17 in autoimmune inflammatory diseases (100) and its established involvement in inducing ventricular arrhythmias in ischemic heart failure (101). In addition, both IL-17 and IL-6 were identified as independent predictors of DD progression in patients with normal left ventricular ejection fraction who underwent invasive hemodynamic assessment (102).

## Conclusions and research agenda

Compelling evidence underscores the pivotal role of inflammation in the development of HFpEF. Endothelial dysfunction emerges as a critical early biomarker, signaling the onset of microvascular damage that can progress to diastolic dysfunction and ultimately HFpEF. Despite these insights, there is a notable absence of clinical trials focused on identifying the optimal diagnostic approach for early detection of DD and stratifying patients for targeted therapeutic protocols based on the type and intensity of underlying inflammation.

No long-term studies have yet evaluated whether tailored treatments can reduce HFpEF incidence in patients with autoimmune chronic inflammatory diseases such as RA, SLE, or SSc. Additionally, the field lacks consensus on key diagnostic thresholds, such as the cutoff values for assessing DD or levels of natriuretic peptides (e.g., NT-proBNP) indicative of imminent HFpEF (103). Research should prioritize defining whether NT-proBNP levels warrant routine annual evaluation, particularly in older patients. The importance of early biomarker evaluation is further highlighted by data from the U.S. National Inpatient Sample Database (2016–2020), which showed that SLE patients hospitalized with acute decompensated heart failure—whether HFpEF or HFrEF—had a mean age of 61 years, compared to 72 years for non-SLE patients. SLE patients also exhibited higher in-hospital mortality rates, emphasizing the need for timely identification of predictive biomarkers to guide early interventions (104).

This approach gains urgency in the context of ACIDs coexisting with metabolic comorbidities such as type 2 diabetes or obesity, particularly in aging populations, where the cumulative risk of HF increases significantly (103). These scenarios reflect the additive impact of metabolic dysfunction and chronic inflammation on cardiac damage. Addressing this, a cardio-immuno-rheumatologic framework should be integrated into clinical practice (105, 106), ensuring that patients with persistent active inflammation are systematically monitored for HFpEF risk.

For diagnostic precision, the H2FPEF score—a composite tool combining clinical and echocardiographic parameters—offers a valuable approach. This scoring system can predict HFpEF with up to 95% probability when the score exceeds 5/9 (Table 3). Implementing such algorithms could revolutionize screening and management strategies in ACIDs, ensuring timely intervention for patients at elevated cardiovascular risk.

**Table 3:** H2FPEF score to evaluate the possible presence of HFpEF in patients with symptomatic dyspnea.

Clinical variables	Points
Weight (BMI > 30)	2
Hypertension (antihypertensive medications)	1
Atrial fibrillation (history or presence)	3
Pulmonary hypertension (RVSP at rest >35 mmHg)	1
Age (age >60 yrs)	1
Filling pressure (Rest E/e’ > 9)	1

Score 0–1	Score 2–5	Score 6–9
HFpEF ruled out	HFpEF possible: assess rest/stress RHC or Echo stress	Very likely HFpEF

BMI, body mass index; RVSP, right ventricle systolic pressure; E/e’, ratio of early diastolic mitral inflow blood velocity to mitral annular tissue velocity; RHC, right heart catheterization.

Future research must focus on:

1. Longitudinal studies evaluating the impact of targeted anti-inflammatory therapies on HFpEF incidence across RA, SLE, and SSc.
2. Establishing evidence-based thresholds for biomarkers like NT-proBNP to guide routine screening.
3. Developing and validating diagnostic algorithms that integrate inflammatory markers, clinical parameters, and imaging data to improve early identification and risk stratification.

By addressing these gaps, we can move closer to a personalized, proactive approach in preventing HFpEF, particularly in high-risk populations.

Finally, considering the heterogeneity of the available studies, particularly regarding HFpEF definitions, patient populations, and outcome measures, as well as the scarcity of randomized controlled trials in autoimmune settings, our conclusions should be interpreted with caution. These limitations further underscore the urgent need for disease-specific, prospective investigations.

## Glossary

HFpEF: Heart Failure with Preserved Ejection Fraction
HFrEF: Heart Failure with Reduced Ejection Fraction
LVEF: Left Ventricular Ejection Fraction
LV: Left Ventricle
DD: Diastolic Dysfunction
ED: Endothelial Dysfunction
IL-6: Interleukin-6
CRP: C-reactive Protein
RA: Rheumatoid Arthritis
SLE: Systemic Lupus Erythematosus
SSc: Systemic Sclerosis
APS: Anti-Phospholipid Syndrome
FMD: Flow-Mediated Dilation
ECM: Extracellular Matrix
TGF-*β*: Transforming Growth Factor Beta
NF-κB: Nuclear Factor Kappa-light-chain-enhancer of activated B cells
AP-1: Activator Protein 1
ROS: Reactive Oxygen Species
eNOS: Endothelial Nitric Oxide Synthase
NO: Nitric Oxide
*α*-SMA: Alpha Smooth Muscle Actin
CCR2: C-C Chemokine Receptor Type 2
STAT3: Signal Transducer and Activator of Transcription 3
AMI: Acute Myocardial Infarction
DM2: Type 2 Diabetes Mellitus
hsCRP: High Sensitivity C-Reactive Protein
MACE: Major Adverse Cardiovascular Events
BNP: B-type Natriuretic Peptide
NT-proBNP: N-terminal pro B-type Natriuretic Peptide
TNF: Tumor Necrosis Factor
OSM: Oncostatin M
BITE: Bispecific T-cell Engager
FDR: False Discovery Rate
WHS: Women’s Health Study
PMR: Polymyalgia Rheumatica
UC: Ulcerative Colitis
IBD: Inflammatory Bowel Disease
CFR: Coronary Flow Reserve
