# Supplementary material for: Autoimmune inflammation as a key risk factor for heart failure with preserved ejection fraction: the different types of inflammation driving to HFpEF
Source: Front Med (Lausanne). 2025 Oct 15;12:1557312. doi: 10.3389/fmed.2025.1557312 (PMC12568492; doi:10.3389/fmed.2025.1557312)
Supplement: Supplementary file 1 [file Data_Sheet_1.PDF]

Gremese et al. Autoimmune inflammation as a key risk factor for heart failure with preserved ejection fraction (HFpEF). The different types of inflammation driving to HFpEF.

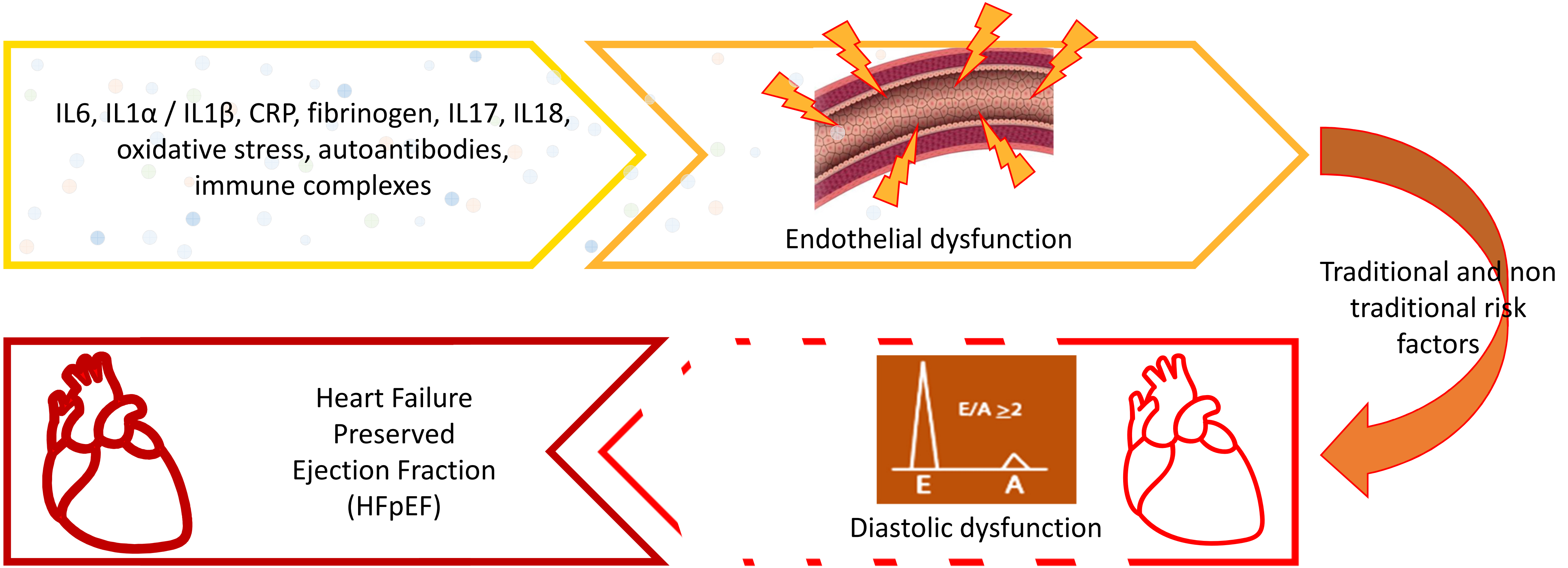

Non traditional risk factors *per se* lead to endothelial dysfunction and along with traditional risk factors (diabetes, hypertension, smoking, dyslipidemia, obesity, life style, age, sex) amplify the risk to end-up with diastolic dysfunction. Diastolic dysfunction represent the pathophysiology of heart failure with preserved ejection fraction (HFpEF).
